# POLB 001, a p38 MAPK inhibitor, decreases local and systemic inflammatory responses following *in vivo* LPS administration in healthy volunteers: a randomised, double-blind, placebo-controlled study

**DOI:** 10.3389/fimmu.2025.1684307

**Published:** 2026-01-23

**Authors:** Digna T. de Bruin, Manon A. A. Jansen, Diana R. Pereira, Lisa van Schijndel, Erica S. Klaassen, Marije E. Otto, Jeremy Skillington, Paula Maguire, Liam Tremble, Alan Bell, Laura Maher, Katsuhiro Mihara, Mark Sumeray, Derek W. Gilroy, Naomi B. Klarenbeek, Matthijs Moerland

**Affiliations:** 1 Centre for Human Drug Research (CHDR), Leiden, Netherlands; 2 Leiden University Medical Center, Leiden, Netherlands; 3 Leiden Academic Centre for Drug Research, Universiteit Leiden, Leiden, Netherlands; 4 Poolbeg Pharma Limited, London, United Kingdom; 5 Department of Experimental and Translational Medicine, University College London Research, London, United Kingdom

**Keywords:** Cytokine release syndrome (CRS), *in vivo*, inflammation, LPS, p38 MAPK

## Abstract

**Background and aim:**

POLB 001 is an oral p38 mitogen-activated protein kinase (MAPK) inhibitor in development for the prevention of cancer immunotherapy-induced cytokine release syndrome (CRS). It has previously been shown to be well tolerated and capable of decreasing *ex vivo* lipopolysaccharide (LPS)-induced tumour necrosis factor (TNF) secretion in a phase 1 first-in-human trial. This study aimed to evaluate the anti-inflammatory effects of POLB 001 following *in vivo* LPS administration in healthy volunteers.

**Methods:**

Participants received POLB 001 at doses of 30, 70, or 150 mg, or placebo, twice daily for seven consecutive days and were challenged locally with intradermal (ID) LPS on day 4 and systemically with intravenous (IV) LPS on day 6. Following ID LPS administration, skin perfusion and erythema were measured, and skin suction blisters were created to collect blister fluid containing infiltrating immune cells and extracellular fluid. Following IV LPS administration, circulating cytokine levels, leukocyte counts, leukocyte p38 MAPK phosphorylation levels, and vital signs were measured.

**Results:**

POLB 001 was well tolerated. It reduced the ID LPS-driven immune cell attraction and cytokine responses measured in blister fluid. The suppression of immune cell recruitment was most pronounced in neutrophils (72.4%–81.5%, *p* = 0.0091), classical monocytes (68.4%–73.6%, *p* = 0.0036), CD3^+^ T cells (56.4%–65.9%, *p* = 0.0047), and myeloid dendritic cells (59%–64.4%, *p* = 0.0174). The suppression of cytokine responses was most pronounced for TNF (35.3%–65.1%, *p* = 0.0099). Overall, POLB 001 did not substantially modulate the intradermal LPS-driven increase in local erythema and perfusion. POLB 001 significantly reduced the IV LPS-driven increase in interleukin (IL)-6, IL-8, and TNF (37.7%–80.7%, all *p* < 0.0003), p38 MAPK phosphorylation levels in target cells (16.7%–60.9%, all *p* < 0.0001*)*, and heart rate increase (4–9.3 bpm, *p* < 0.0001).

**Conclusion:**

POLB 001 was safe and well-tolerated. Pharmacodynamic findings confirm that POLB 001 inhibits LPS-induced local and systemic inflammation *in vivo* through inhibition of p38 MAPK.

**Clinical trial registration:**

https://onderzoekmetmensen.nl/en/trial/51741, identifier NL81214.056.22.

## Introduction

Mitogen-activated protein kinases (MAPKs) are components of signalling cascades that initiate inflammatory cellular responses when activated by extracellular stimuli (1). Several subgroups of MAPKs have been identified, including p38 MAPK. Triggers for p38 MAPK activation include lipopolysaccharide (LPS) and other bacterial products, ultraviolet irradiation, hypoxia, and inflammatory cytokines (1, 2). Activation of the p38 signalling pathway plays an important role in the production of various inflammatory mediators, such as tumour necrosis factor (TNF) (3, 4). Inhibition of the p38 MAPK signalling pathway results in reduced production of these inflammatory mediators.

POLB 001 (previously known as Org 48775-0) is a potent and highly selective p38α/β MAPK inhibitor in development for cancer immunotherapy-induced cytokine release syndrome (CRS). POLB 001 has shown efficacy in several pharmacological disease models, including humanised mouse models of CRS (5). It was well tolerated under both fed and fasted conditions in a previous phase 1 trial in healthy male volunteers. In that first-in-human single ascending dose (SAD) study and a multiple ascending dosing (MAD) study, POLB 001 was administered as a single dose up to 600 mg and as multiple doses twice daily for six consecutive days, up to 150 mg. Using peripheral whole blood samples from treated participants, POLB 001 was shown to effectively decrease LPS-induced TNF release (6).

While previous results demonstrate that POLB 001 decreases LPS-induced TNF release from circulating leukocytes *ex vivo*, the effects of POLB 001 on *in vivo* inflammatory responses were unknown. *Ex vivo* refers to the evaluation of drug activity in a test tube, with the immune trigger added to the biospecimen obtained from drug-exposed participants. In contrast, *in vivo* refers to the evaluation of drug activity in humans, where drug-treated participants were exposed to the immune trigger. The aim of this phase 1 study was to determine the inhibitory effects of POLB 001 on the inflammatory response following local and systemic inflammation in healthy volunteers, upon intradermal (ID) and intravenous (IV) LPS administration. A multimodal pharmacodynamic evaluation approach was employed, comprising clinical endpoints, imaging-based endpoints, local tissue response endpoints, and blood-based endpoints.

## Materials and methods

### Study design and volunteer inclusion

This was a randomised, double-blind, placebo-controlled, parallel-group, MAD study in healthy volunteers. The study was executed at the Centre for Human Drug Research, the Netherlands, between August 2022 and December 2022. It adhered to ethical principles derived from international guidelines, including the Declaration of Helsinki, as well as all applicable ICH, GCP, and Dutch Medical Research Involving Human Subjects Act guidelines. The study protocol was registered in the EudraCT database (number 2022-001458-48). The protocol (registered at Toetsingonline, number NL81214.056.22) was approved by a medical ethics committee (Stichting Beoordeling Ethiek Biomedisch Onderzoek, Assen, the Netherlands) prior to the start of the clinical phase. All participants provided written informed consent before any study-related activities were performed.

The study aimed to recruit 36 healthy volunteers. Volunteers were eligible if they were men, had Fitzpatrick skin type I–III, and were aged between 18 and 55 years. Male volunteers were excluded if any of the following criteria applied: previous participation in a systemic (IV or inhaled) LPS challenge study within a year before the first study day, a current or recurrent clinically significant skin condition at the intradermal LPS target areas (including tattoos), or a known immunodeficiency.

**Figure 1:**
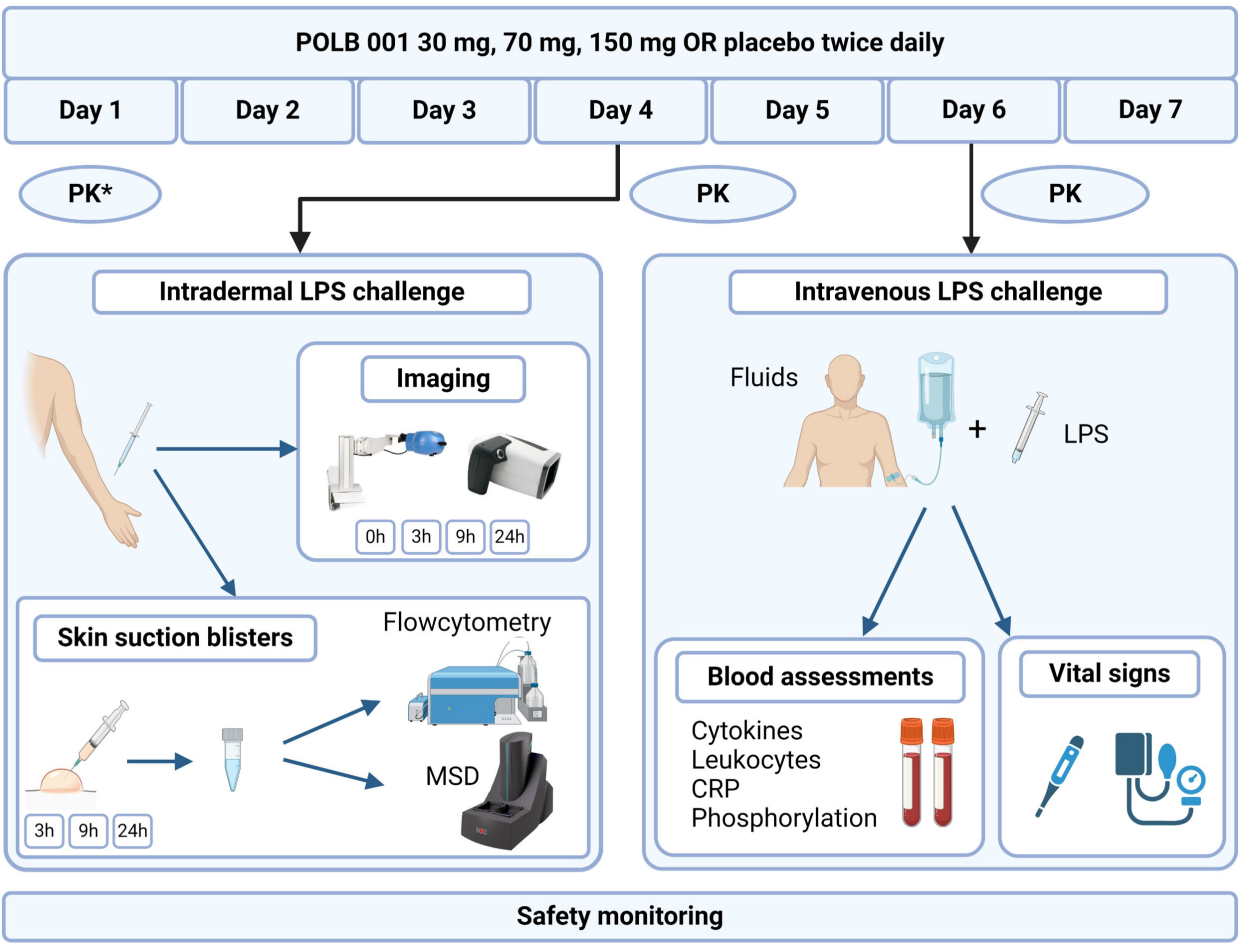
Overview of the study design. POLB 001 or a matching placebo was administered twice daily for seven consecutive days. On day 4, participants received four intradermal injections of LPS. The local response was evaluated at specified timepoints by using different imaging techniques and skin blister fluid analysis. On day 6, participants received a single dose of intravenous LPS, with pre- and posthydration. Endpoints included different blood assessments and vital signs. The asterisk indicates PK on day 1 only for the highest dose group. PK, pharmacokinetics; LPS, lipopolysaccharide; MSD, Meso Scale Discovery; CRP, C-reactive protein. *Created with**Biorender.com*.

Participants received oral doses of POLB 001 (30 mg—low dose, 70 mg—intermediate dose, 150 mg—high dose) or a matching placebo twice daily for seven consecutive days (days 1 to 7) (**Figure 1**). Participants were scheduled to take the study medication either at the study site or at home. Drug intake at home was monitored using an electronic Patient Reported Outcomes (ePRO) application. All participants received the ID challenge on day 4 and the IV LPS challenge on day 6 upon admission to the clinical unit. Safety monitoring was performed throughout the study course for all participants. Blood samples for PK serum analysis were collected predose and repeatedly on days 1, 4, and 6, and were analysed at the Ardena Bioanalytical Laboratory (Assen, the Netherlands).

### Investigational drug, placebo, and challenge agent

Both the study medication and LPS (for both ID and IV administration) were formulated by the pharmacy at the Leiden University Medical Centre, Leiden, the Netherlands. POLB 001 was administered as an oral suspension of 30, 70, or 150 mg POLB 001 powder in 20 mL SyrSpend^®^ Cherry SF PH4 Vehicle. For each dose level, a matching placebo was prepared. Participants were instructed to fast for 8 h prior to every morning dose and for 1 h prior to each evening dose. Purified LPS, prepared from *Escherichia coli* O113:H10:K negative (US Standard Reference Endotoxin), was used as a challenge agent. The LPS batch was manufactured in the United States (USA) by List Biological Laboratories (Campbell, California). Each participant received four ID LPS injections on the volar forearms, each consisting of 5 ng LPS in 0.05 mL of 0.9% sodium chloride (NaCl) solution (a total of 20 ng per participant). A different LPS injection site was used for each blister timepoint. Participants also received an IV infusion of glucose (2.5% w/v) and NaCl (0.45% w/v) solution, starting 2 h prior to LPS administration and continuing for 6 h afterward. IV LPS consisted of 1 ng/kg LPS in 10 mL of 0.9% NaCl solution, administered as a 2-min infusion (7).

### Evaluation of *ex vivo* pharmacodynamic effects

For the evaluation of *ex vivo* pharmacodynamic effects, LPS was added as an immune trigger to test tubes containing whole blood obtained from drug-exposed participants. Baseline whole blood samples were collected on days 1 and 6 prior to the IV LPS challenge. Blood was diluted (1:1) with RPMI 1640 supplemented with 25 mM HEPES and l-glutamine, and stimulated with LPS (2.5 ng/mL). Samples were incubated for 24 h at 37 °C (5% CO_2_), and the supernatant was stored. Secreted interleukin (IL)-1β, IL-6, IL-8, and TNF were analysed from the supernatant using a Meso Scale Discovery (MSD) multiplex immunoassay (Ardena, Assen, the Netherlands).

### Evaluation of the intradermal LPS response

#### Skin imaging

The skin was imaged pre-LPS and at 3, 9, and 24 h afterward. Perfusion was evaluated using laser speckle contrast imaging (LSCI) (PeriCam PSI System, Perimed Jäfälla, Sweden), and erythema was assessed with multispectral imaging using Antera 3D (Miravex, Dublin, Ireland). The performance and analysis of these noninvasive measurements were standardised for all participants.

#### Suction blister induction and analysis

After skin imaging was performed, 10 mm suction blisters were generated over the LPS-injected skin areas, as previously reported (8). Three suction blisters were created per participant: at 3, 9, and 24 h after the ID LPS injections.

Blister fluid was collected in a plate containing 50 μL of 3% cold sodium citrate (Sigma‐Aldrich, St. Louis, MO, USA) in PBS (Gibco, Thermo Fischer Scientific, Waltham, MA, USA). Levels of IL-1β, IL-6, IL-8, and TNF in blister fluid were analysed by MSD (Ardena, Assen, the Netherlands). Leukocytes in blister fluid were immunophenotyped by flow cytometry (for antibodies, see **Supplementary Table S1**). The gating strategy for blister leukocytes was selected using a peripheral blood sample collected before LPS administration (see **Supplementary Figure S1**). Flow cytometry measurements were done in the MACSQuant Analyzer 16 (Miltenyi Biotec GmbH, Bergisch Gladbach, Germany), and data were analysed with Flowlogic 8.7 (Inivai Technologies, Mentone, VIC, Australia) at the Centre for Human Drug Research (CHDR) laboratory (Leiden, the Netherlands).

### Evaluation of the intravenous LPS response

#### p38 MAPK phosphorylation in circulating leukocytes

Blood samples for p38 MAPK phosphorylation analyses were collected prior to IV LPS administration and at 1, 1.5, and 2 h afterward. Within 1 h of collection, sodium heparinised whole blood was fixed, and red blood cell lysis was performed simultaneously. Cells were permeabilised and stained with anti-CD14 (REA 599) and p38 MAPK antibody (RUO 36/p38 [pT180/pY182]) in the same test tube (see **Supplementary Table S1**). Live cells were gated from the forward scatter (FSC) vs. side scatter (SSC) leukocyte population, and doublets were removed by gating for FSC-Height (FSC-H) vs. FSC-Area (FSC-A) (**Supplementary Figure S2**). Monocytes were identified as CD14^+^. Phosphorylation levels of p38 MAPK in whole blood and in CD14^+^ cells were expressed as a percentage and median fluorescence intensity (MFI). Analysis was performed at the laboratory of CHDR (Leiden, the Netherlands).

#### Clinical signs and symptoms

Vital sign measurements, including heart rate, blood pressure, and temperature, were measured to evaluate the clinical response to IV LPS.

#### Circulating cytokines

Blood was collected from each participant prior to IV LPS administration and 1–4 h (every hour), 6 h, and 9 h after IV LPS administration for analysis of circulating cytokine levels by MSD: IL-1β, IL-6, IL-8, IL-10, and TNF (Ardena, Assen, the Netherlands).

#### Leukocyte differentiation and C-reactive protein

Blood samples for leukocyte differential analysis were collected prior to the IV LPS administration and at 3, 6, and 24 h after LPS administration. Blood samples for C-reactive protein (CRP) analysis were collected prior to the IV LPS challenge and 6, 9, and 24 h after administration. All blood samples were analysed at the laboratory of the Leiden University Medical Center (“Klinische chemie en laboratoriumgeneeskunde” LUMC, Leiden, the Netherlands).

### Statistics

A sample size of nine per treatment group was deemed sufficient based on historical data (8–10). This sample size provides at least 80% power to detect a 50% decrease in LPS-induced systemic and dermal responses using a two-sided significance level of 0.05. Repeatedly measured data were analysed with a mixed model analysis of covariance (ANCOVA) with treatment, time, and treatment-by-time as fixed factors, subject as a random factor, and the (average) baseline measurement as a covariate. Treatment effects were calculated for each parameter over the time period from baseline up to 24 h, except for p38 MAPK phosphorylation (up to 2 h postdose), *ex vivo* analysis (baseline day 1 vs. day 6 pre-IV LPS), and circulating cytokines (up to 9 h postdose). The following comparisons were calculated for both the ID and IV LPS challenges (analysed separately): POLB 001 at 30 mg vs. pooled placebo, POLB 001 at 70 mg vs. pooled placebo, and POLB 001 at 150 mg vs. pooled placebo. Comparisons were reported with estimated differences, 95% confidence intervals (CI), and *p*-values. All calculations were performed using SAS for Windows V9.4 (SAS Institute Inc., Cary, NC, USA).

## Results

Sixty-three participants were screened for this study. Of these, 41 participants, including reserve participants, were enrolled. Thirty-six participants were treated in three cohorts of 12 participants each (nine active vs. three placebo).

### Baseline characteristics

All 36 participants were men, and 94.4% were White. The mean ± SD age was 28.6 years ± 10.1 years. Further baseline characteristics are presented in **Table 1**.

**Table 1:** Baseline demographic characteristics.

Treatment groups	All participants	POLB 001 at 30 mg	POLB 001 at 70 mg	POLB 001 at 150 mg	Placebo
Age (years)					
*N*	36	9	9	9	9
Mean (SD)	28.6 (10.1)	32.6(11.6)	27.0 (9.8)	24.4 (4.7)	30.4 (12.2)
Median	25	26	25	22	25
Min, Max	18, 55	23, 52	18, 51	21, 36	21,55
Height (cm)					
*N*	36	9	9	9	9
Mean (SD)	182.03 (6.82)	183.18 (7.12)	181.86 (8.05)	182.20 (7.20)	180.87 (5.75)
Median	181.6	182.2	181.1	183.4	181.2
Min, Max	170.1, 198.2	172.6, 193.7	170.1, 198.2	172.0, 194.6	172.7, 189.2
Weight (kg)					
*N*	36	9	9	9	9
Mean (SD)	75.871 (9.929)	78.900 (8.530)	75.144 (12.744)	73.433 (7.633)	76.006 (10.967)
Median	74.25	78.20	74.65	70.65	73.85
Min, Max	56.60, 100.20	71.95, 99.50	56.60, 100.20	64.05, 86.20	63.00, 94.75
BMI (kg/m^2^)					
*N*	36	9	9	9	9
Mean (SD)	22.90 (2.83)	23.59 (2.93)	22.71 (3.72)	22.09 (1.23)	23.22 (3.11)
Median	22.8	23.6	22.1	22.3	23.1
Min, Max	18.8, 30.7	21.3, 30.7	18.8, 30.6	19.5, 23.5	19.2, 28.7
Sex (*N* [%])					
Male	36 (100%)	9 (100%)	9 (100%)	9 (100%)	9 (100%)
Race (*N* [%])					
Asian	1 (2.8%)	0 (0%)	1 (11.1%)	0 (0%)	0 (0%)
Other	1 (2.8%)	0 (0%)	0 (0%)	1 (11.1%)	0 (0%)
White	34 (94.4%)	9 (100%)	8 (88.9%)	8 (88.9%)	9 (100%)

SD, standard deviation.

### Safety and tolerability

Administration of POLB 001 for seven consecutive days was well tolerated. No severe adverse events (SAEs) occurred following dosing. No meaningful relationships between POLB 001 dose and the incidence of adverse events (AEs) were observed. No clinically relevant changes in ECG parameters, blood chemistry, or haematology—apart from CRP and leukocyte differential levels described as PD endpoints and an increase in liver enzymes in one participant—were observed.

A full list of AEs can be found in **Supplementary Table S2**. Headache was the most commonly reported AE overall and in the POLB 001 treatment groups. Dizziness, fatigue, and chills were additional commonly reported AEs in all treatment groups. Only three participants who received POLB 001 reported events considered probably related to the study drug. Most AEs were possibly or unrelated to POLB 001 and could be related to the LPS challenges.

### Pharmacokinetics

POLB 001 showed dose-proportional PK for *C*_max_ and exposure over 12 h (AUC_12 h_), in line with the previous study (6).

### *Ex vivo* pharmacodynamics

LPS-induced IL-1β, IL-6, IL-8, and TNF release *ex vivo* was decreased by POLB 001 in the day 6 samples compared to baseline. Percentages of inhibition by POLB 001 ranged from 38.8% to 42.2% for IL-1β, 32.1% to 52.2% for IL-6, 52.1% to 54.7% for IL-8, and 53% to 59.8% for TNF (**Supplementary Figure S3**).

### *In vivo* pharmacodynamics: ID LPS challenge response

#### POLB 001 does not impact LPS-driven elevation in skin erythema and perfusion

**Figure 2:**
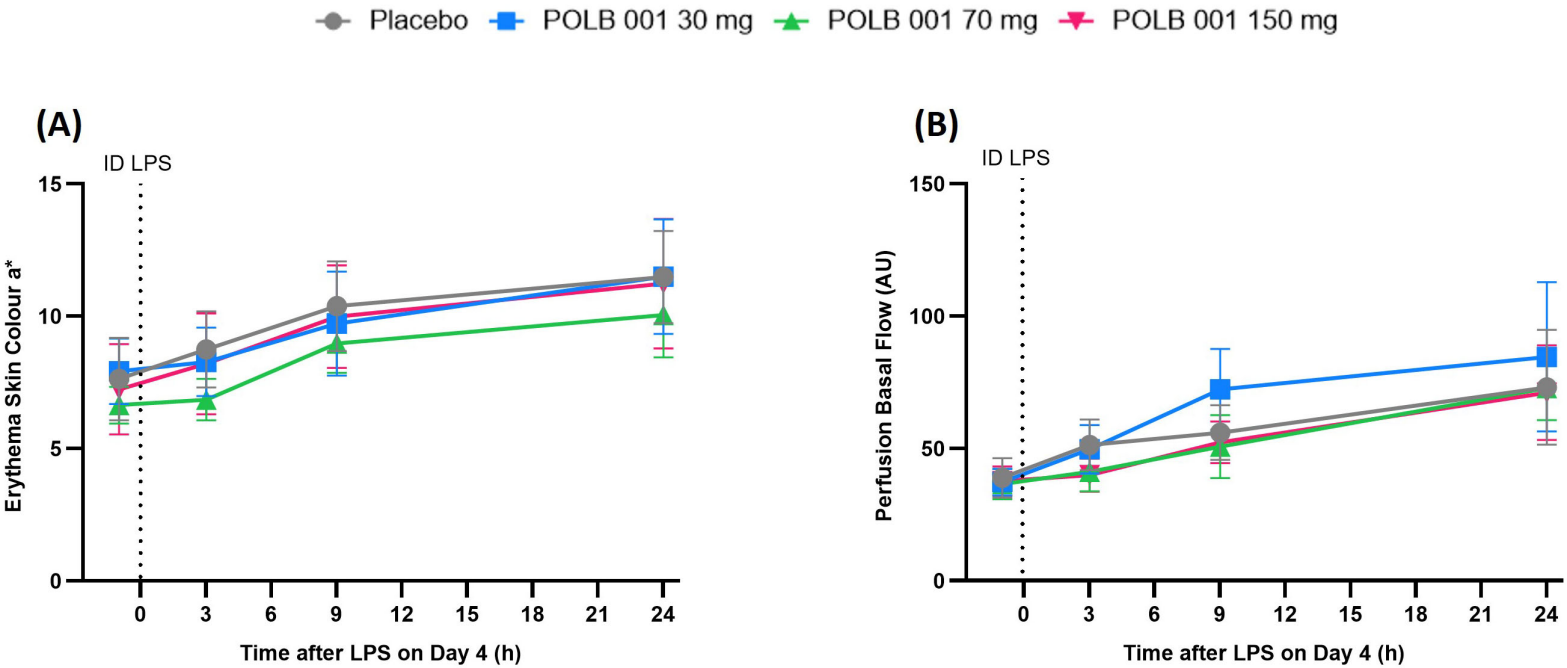
Quantification of the inflammatory skin response following the intradermal LPS challenge by **(A)** multispectral imaging (erythema) and **(B)** laser speckle contrast imaging (LSCI; perfusion). In **(A)**, POLB 001–30 mg vs. placebo showed *p* < 0.05, whereas other contrasts showed *p* > 0.05. In **(B)**, POLB 001 vs. placebo, all *p* > 0.05. Data are expressed as means with SD. AU, arbitrary units; ID, intradermal; LPS, lipopolysaccharide.

After administration of ID LPS, a gradual increase in skin erythema and perfusion was observed up to 24 h (**Figure 2**). POLB 001 at 30 mg slightly but significantly decreased LPS-driven erythema compared to placebo (change: − 0.6782 Antera^®^ skin colour a* units [redness; *p* < 0.05]). However, the POLB 001 at 70 and 150 mg dose groups showed no significant effect compared to placebo (change: − 0.5495 and 0.0068 Antera^®^ skin colour a* units with *p* = 0.1040 and *p* = 0.9831, respectively). Consistent with this, POLB 001 did not significantly reduce the LPS-driven increase in skin perfusion compared to placebo.

#### POLB 001 suppresses LPS-driven immune cell attraction and cytokine responses in blister fluid

**Figure 3:**
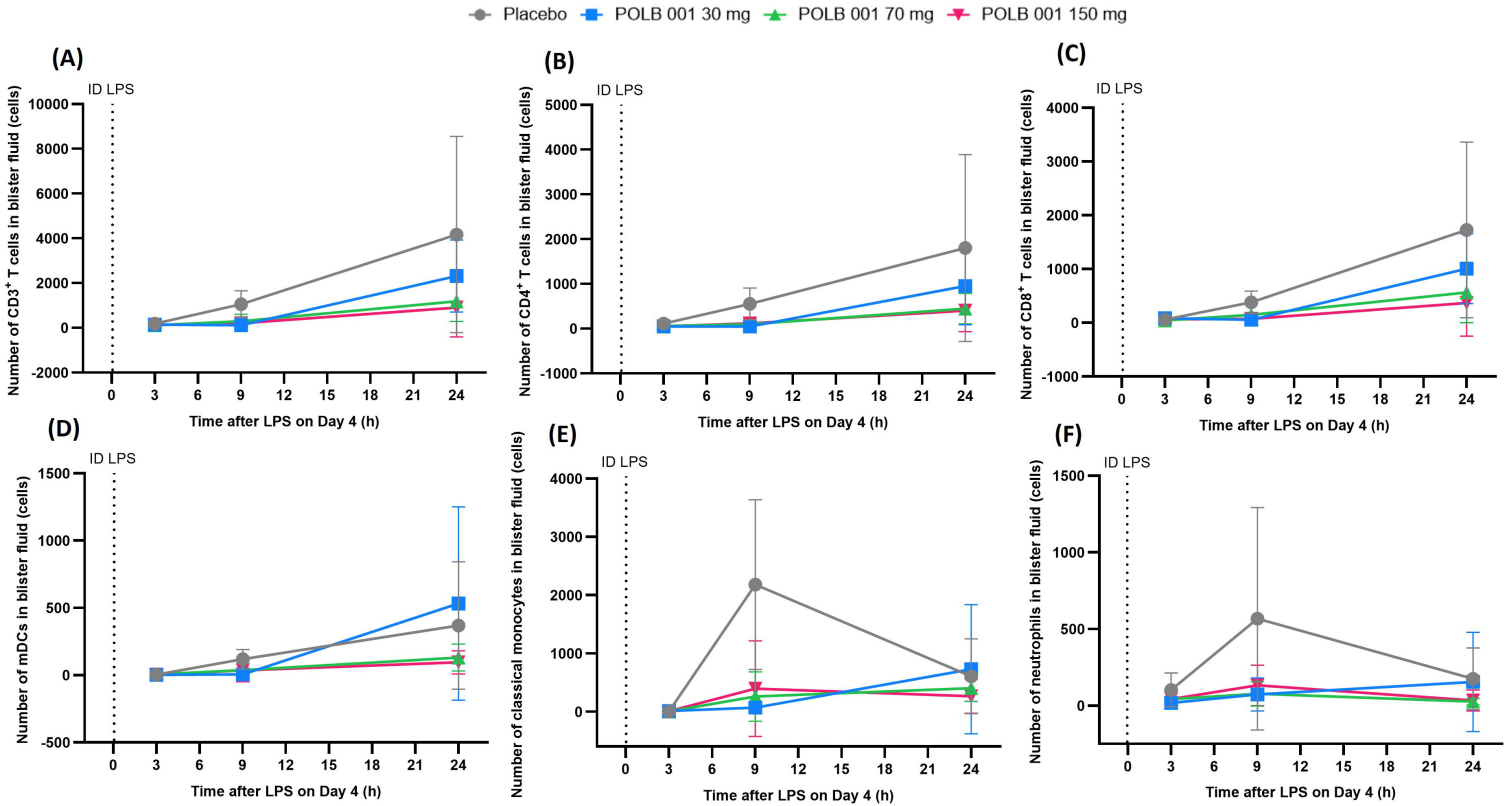
Overview of skin blister fluid analysis by flow cytometry following intradermal LPS administration: **(A)** CD3^+^ T cells, **(B)** CD4^+^ T cells, **(C)** CD8^+^ T cells, **(D)** mDCs, **(E)** classical monocytes, and **(F)** neutrophils. In **(A**, **B)**, POLB 001 vs. placebo, all *p* < 0.01. In **(C)**, POLB at 30 mg vs. placebo showed *p* > 0.05, whereas POLB 001 at 70 and 150 mg vs. placebo showed *p* < 0.01. In **(D)**, POLB 001 vs. placebo, all *p* < 0.05. In **(E)**, POLB 001 vs. placebo, all *p* < 0.01. In **(F)**, POLB 001 vs. placebo, all *p* < 0.05. Data are expressed as means with SD. ID, intradermal; LPS, lipopolysaccharide; mDCs, myeloid dendritic cells; SD, standard deviation.

**Figure 4:**
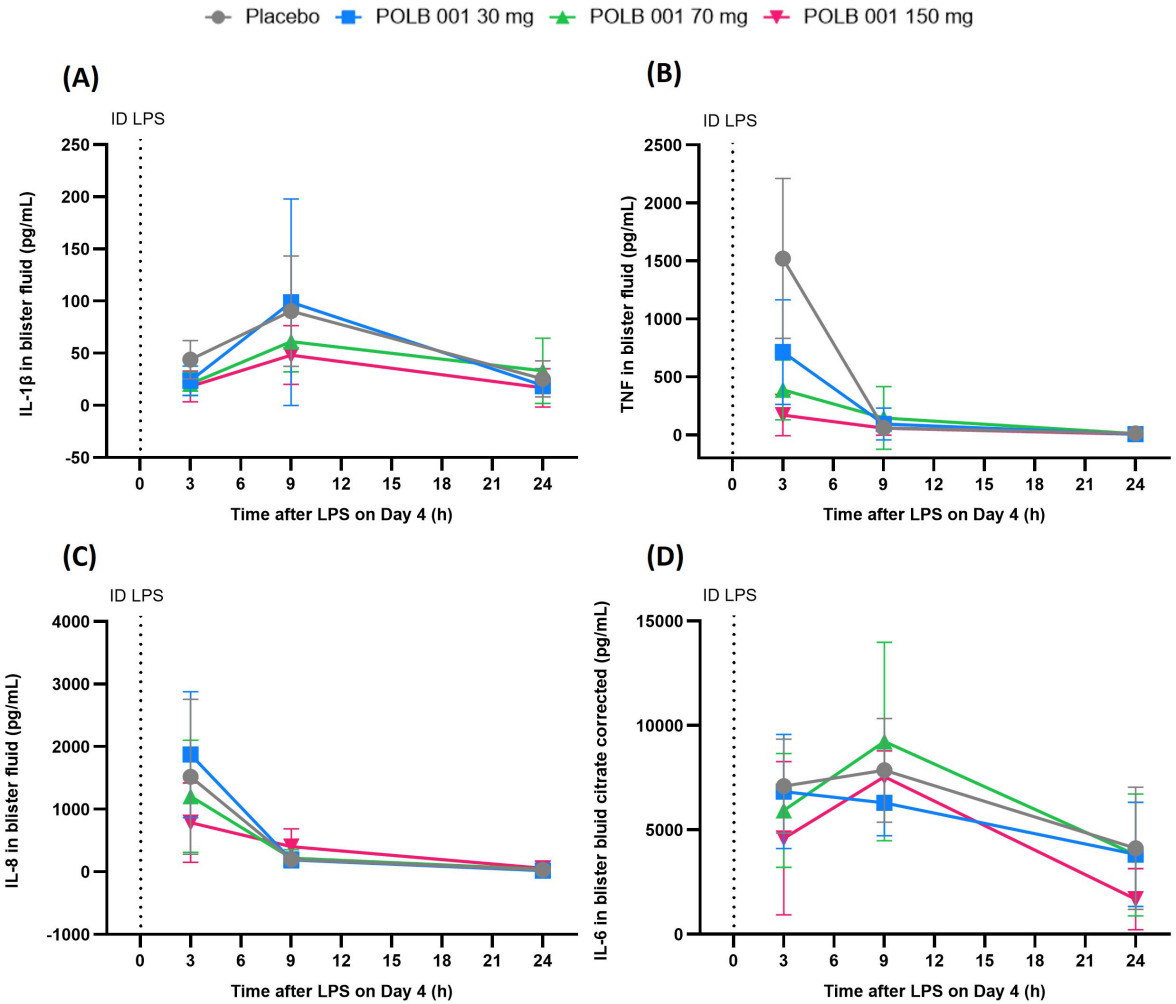
Overview of cytokine responses in skin blister fluid following intradermal LPS administration: **(A)** IL-1β response, **(B)** TNF response, **(C)** IL-8 response, and **(D)** IL-6 response. In **(A)**, POLB 150 mg vs. placebo showed *p* < 0.02, whereas other contrasts showed *p* > 0.05. In **(B)**, POLB 150 mg vs. placebo showed *p* < 0.02, whereas other contrasts showed *p* > 0.05. In **(C)**, POLB 001 vs. placebo, all *p* > 0.05. In **(D)**, due to too many values above the ULOQ, the requirement for analysis was not met. Data are expressed as means with SD. *ID*, intradermal; LPS, lipopolysaccharide; TNF, tumour necrosis factor; IL, interleukin.

In the absence of POLB 001, ID LPS administration led to an increase in neutrophils and classical monocytes, peaking at 9 h, and an increase in myeloid dendritic cells (mDCs), plasmacytoid DCs (pDCs), CD3^+^ T cells, CD4^+^ T cells, CD8^+^ T cells, nonclassical monocytes, intermediate monocytes, B cells, natural killer (NK) cells, and total immune cells, peaking at 24 h in blister fluid (**Figure 3**, **Supplementary Figure S4**). For cytokines, a rapid response of IL-8 and TNF was observed, peaking at 3 h, whereas a more delayed response was observed for IL-1β and IL-6, peaking at 9 h (**Figure 4**).

Treatment with POLB 001 resulted in a decrease in the number of CD3^+^ T cells, CD4^+^ T cells, mDCs, classical monocytes, neutrophils, and total immune cells in blister fluid compared with placebo treatment (**Figures 3A, B, D–F**, **Supplementary Figure S4A**). This inhibition was dose-dependent for CD3^+^ T cells, classical monocytes, and total immune cells, with percentages of suppression ranging from 56.4% to 65.9% (all dose groups *p* < 0.01 vs. placebo) for CD3^+^ T cells, 68.4% to 73.6% (all dose groups *p* < 0.01 vs. placebo) for classical monocytes, and 65.5% to 70.9% (all dose groups *p* < 0.01 vs. placebo) for total immune cells. For mDCs, CD4^+^ T cells, and neutrophils, percentages of inhibition compared with placebo were comparable across the three dose levels: 59.0% to 64.4% for mDCs (all dose groups *p* < 0.05 vs. placebo), 62.8% to 66.2% for CD4^+^ T cells (all dose groups *p* < 0.01 vs. placebo), and 72.4% to 81.5% for neutrophils (all dose groups *p* < 0.05 vs. placebo). Additionally, CD8^+^ T cells and NK cells were reduced by POLB 001 treatment (**Figure 3C**, **Supplementary Figure S4B**), with significant inhibition observed for POLB 001 at 70 and 150 mg compared to placebo (64.1% and 66.6% for CD8^+^ T cells, 61.9% and 63.0% for NK cells, respectively; all dose groups *p* < 0.01 vs. placebo). For pDCs, nonclassical monocytes, intermediate monocytes, and B cells, there were too many zero values to allow formal analysis, mainly due to (i) a biologically delayed response to ID LPS by particular cell types, (ii) overall low cell counts, and/or (iii) substantially decreased infiltration induced by POLB 001. However, graphical presentations of nonclassical monocytes, intermediate monocytes, and B cells showed an apparent inhibitory effect of POLB 001 compared to placebo, which appeared to be dose-dependent for nonclassical and intermediate monocytes (**Supplementary Figures S4C–E**). Graphical presentation of pDCs also showed an apparent inhibitory effect with the two highest dose groups (POLB 001 at 70 and 150 mg) at 24 h compared to the placebo group (**Supplementary Figure S4F**).

POLB 001 at 150 mg significantly decreased LPS-driven IL-1β (47.3%, *p* < 0.02 vs. placebo) and TNF (65.1%, *p* < 0.001 vs. placebo) responses in blister fluid compared to placebo (**Figures 4A, B**). No significant effect of POLB 001 compared to placebo was observed for IL-8 (**Figure 4C**). For IL-6, the requirements for analysis were not met due to too many values above the upper limit of quantification (ULOQ), mostly in the placebo group (**Figure 4D**).

### *In vivo* pharmacodynamics: POLB 001 effects on IV LPS challenge response

#### POLB 001 decreases LPS-driven p38 MAPK phosphorylation

**Figure 5:**
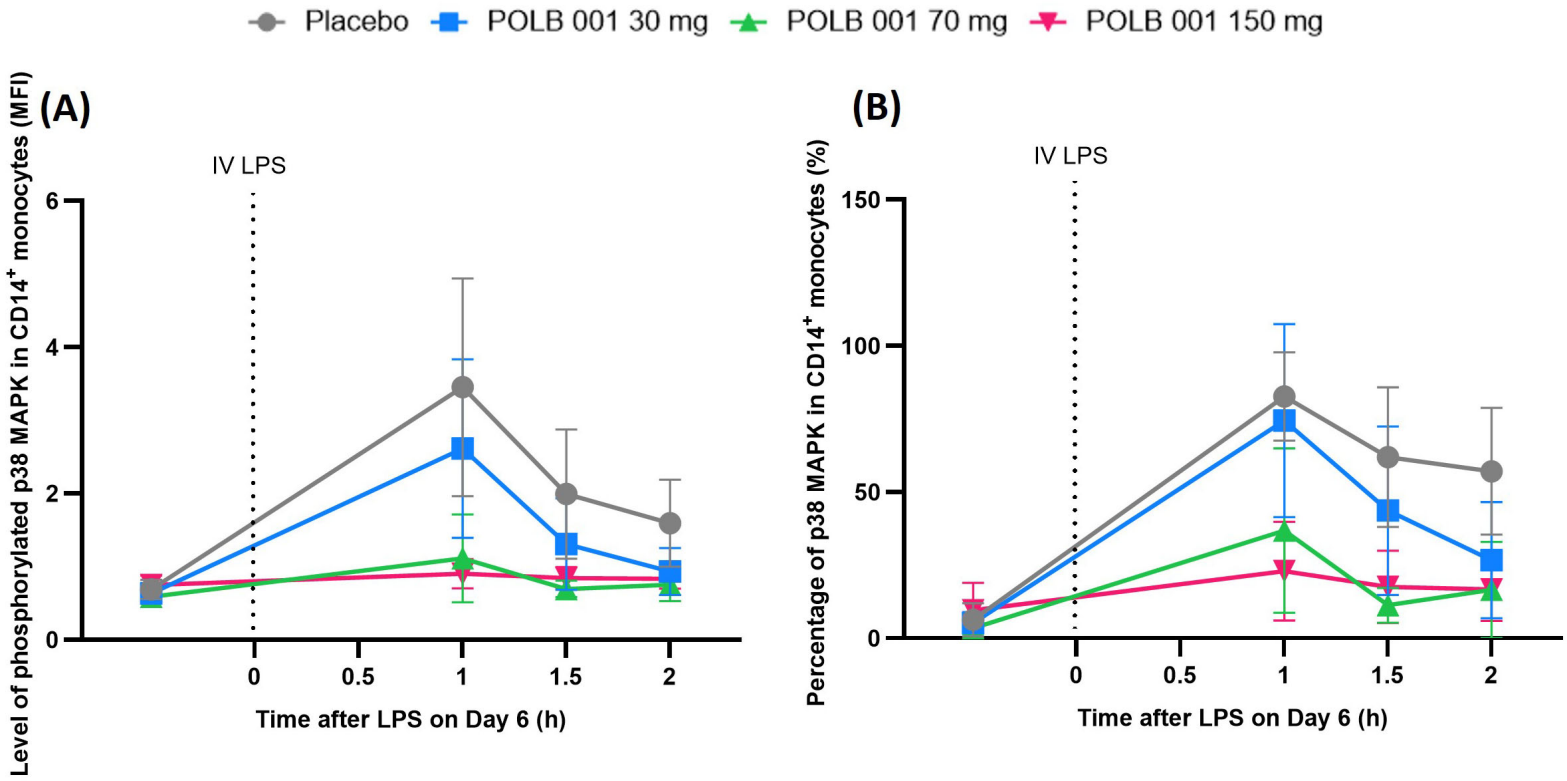
Overview of p38 MAPK phosphorylation in CD14^+^ monocytes expressed as **(A)** MFI and **(B)** percentage. In **(A)**, POLB 001 vs. placebo, all *p* < 0.02. In **(B)**, POLB 001 vs. placebo, all *p* < 0.05. Data are expressed as means with SD. IV, intravenous; LPS, lipopolysaccharide; MAPK, mitogen-activated protein kinase; MFI, mean fluorescence intensity.

IV LPS administration resulted in the phosphorylation of p38 MAPK in whole blood and CD14^+^ monocytes, peaking at 1 h in the placebo group (**Figure 5** for CD14^+^ monocytes; whole blood data not shown).

The level of p38 MAPK phosphorylation in whole blood upon IV LPS challenge was significantly lower in all participants receiving POLB 001, with an inhibition range of 16.7%–28.1% (all dose groups *p* < 0.01 vs. placebo), although no clear dose response was observed. LPS-induced p38 MAPK phosphorylation in CD14^+^ monocytes was significantly and dose-dependently decreased by POLB 001, with an inhibition range of 32.0%–60.9% (all dose groups *p* < 0.02 vs. placebo) compared to the placebo group.

#### POLB 001 suppresses the LPS-driven increase in heart rate without affecting temperature and blood pressure

**Figure 6:**
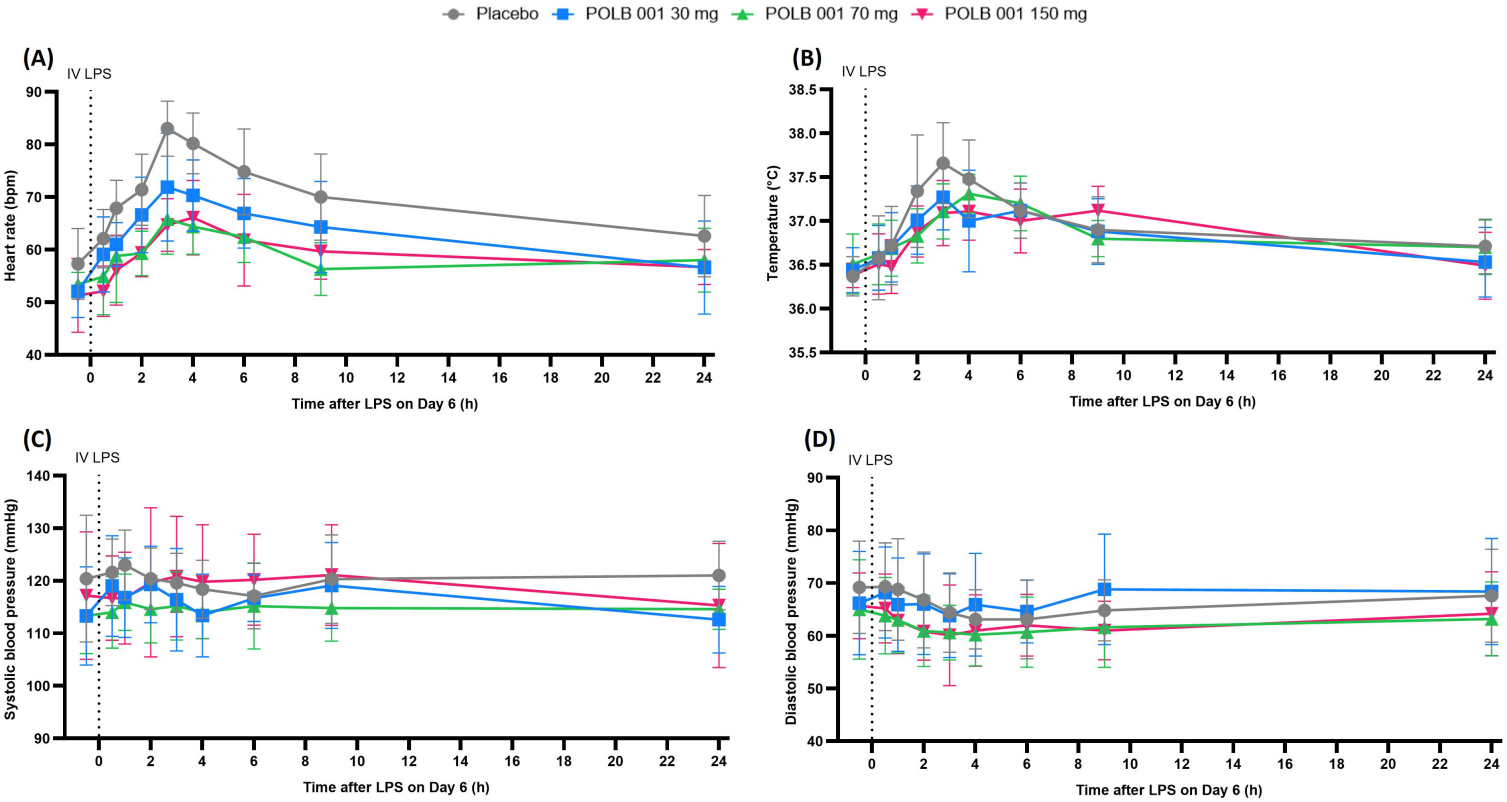
Overview of vital sign measurements during the IV LPS challenge: **(A)** heart rate, **(B)** temperature, **(C)** systolic blood pressure, and **(D)** diastolic blood pressure. In **(A)**, POLB 001 vs. placebo, all *p* < 0.05. In **(B**–**D)**, POLB 001 vs. placebo, all *p* > 0.05. Data are expressed as means with SD. IV, intravenous; LPS, lipopolysaccharide; bpm, beats per minute; °C, degrees Celsius.

Heart rate (HR) increased in all participants as expected following IV LPS, peaking at 3–4 h post-LPS (**Figure 6A**). The increase in HR was 4.0–9.3 beats per minute (bpm) lower in participants receiving POLB 001 compared to placebo (*p* < 0.05 for POLB 001 at 30 mg vs. placebo and *p* < 0.001 for the two highest dose groups vs. placebo). Maximal inhibition was reached for POLB 001 at 70 mg. An increase in body temperature following IV LPS was also observed in all participants (**Figure 6B**), whereas minimal mean changes in blood pressure were noted (**Figures 6C, D**). POLB 001 had no statistically significant effect on LPS-driven changes in body temperature or blood pressure.

#### POLB 001 suppresses the LPS-driven increase in cytokines

**Figure 7:**
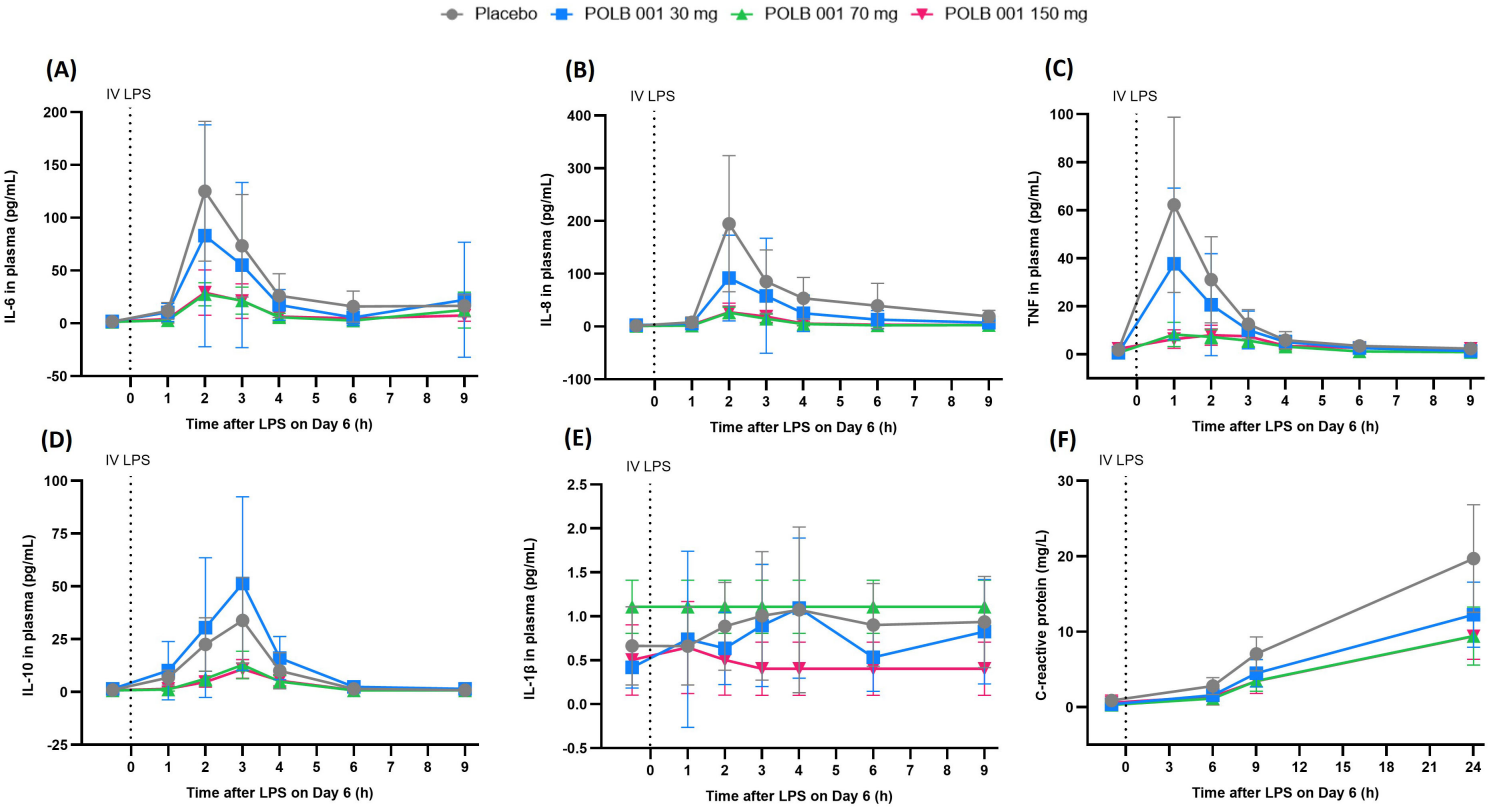
Overview of the cytokine and CRP response in plasma during the IV LPS challenge: **(A)** IL-6, **(B)** IL-8, **(C)** TNF, **(D)** IL-10, **(E)** IL-1β, and **(F)** CRP. In **(A–C)**, POLB 001 vs. placebo, all *p* < 0.05. In **(D)**, POLB 001 at 30 mg vs. placebo showed *p* > 0.05, whereas POLB 001 at 70 and 150 mg vs. placebo showed *p* < 0.001. In **(E)**, due to levels below the LLOQ, the requirements for analysis were not met. In **(F)**, POLB 001–30 at mg vs. placebo showed *p* > 0.05, whereas POLB 001 at 70 and 150 mg vs. placebo showed *p* < 0.05. Data are expressed as means with SD. IV, intravenous; LPS, lipopolysaccharide; TNF, tumour necrosis factor; IL, interleukin; CRP, C-reactive protein.

In the placebo group, IV LPS caused a rapid increase in IL-6, IL-8, TNF, IL-10, and IL-1β in plasma, peaking 1–4 h after administration (**Figures 7A–E**). An increase in CRP levels was also observed, peaking at 24 h (**Figure 7F**).

Treatment with POLB 001 resulted in a significant, dose-dependent reduction in the LPS-driven IL-6 response, with an inhibition range of 37.7%–63.5% compared with placebo (all dose groups *p* < 0.05 vs. placebo). A significant reduction in the LPS-driven increase in IL-8 and TNF in plasma was observed in all POLB 001 treatment groups compared with the placebo group (all dose groups *p* < 0.05 vs. placebo). Maximal inhibition appeared to be reached at a dose of 70 mg, with 80.7% for IL-8 and 73.5% for TNF. For IL-10, a significant inhibition was observed for POLB 001 at 70 mg (62.4%) and 150 mg (62.7%) compared with placebo (both *p* < 0.001 vs. placebo). IL-1β levels in plasma were below the LLOQ, and the data distribution was not normal or log-normal; therefore, the requirements for analysis were not met.

POLB 001 treatment dose-dependently decreased the LPS-driven CRP response, reaching statistical significance for the intermediate dose group, with a decrease of 33.1%, and for the highest dose group, with a decrease of 33.3%, compared with the placebo group (*p* < 0.05 vs. placebo).

#### POLB 001 modulates LPS-driven changes in leukocyte subset levels

Administration of IV LPS resulted in a transient increase in neutrophils and a biphasic response in monocytes (an initial decrease at 3 h followed by an increase at 6 h), as observed in placebo participants (**Supplementary Figure S5**). Moreover, a mild decrease in lymphocytes, eosinophils, and basophils was observed following IV LPS administration (data not shown).

The LPS-driven drop in monocytes was suppressed by POLB 001 in all treatment groups compared to the placebo group (30.9%–52.4%, all dose groups *p* < 0.05 vs. placebo). This effect was not dose-dependent. Neutrophils following IV LPS administration were significantly higher in the intermediate POLB 001 dose group compared to the placebo group (22.5%, *p* < 0.05). For eosinophils, basophils, and lymphocytes, no significant effect of POLB 001 compared with placebo was observed.

## Discussion

The main goal of this randomised controlled trial was to evaluate the effect of POLB 001 on local and systemic inflammatory responses following *in vivo* LPS administration in healthy volunteers. In addition, the safety, tolerability, and PK of POLB 001 were assessed. The doses of POLB 001 administered in this study matched those tested in the previous MAD study (30, 70, and 150 mg twice daily).

POLB 001 was well tolerated, and no treatment-related SAEs or death occurred. The safety profile observed in this study was similar to that reported in the phase 1 study (6), with headache, chills, and dizziness being the most commonly reported AEs. No trends in AEs were observed for POLB 001. As other p38 MAPK inhibitors have been associated with liver enzyme elevations, special attention was paid to increases in liver enzymes, particularly Alanine aminotransferase (ALAT). One participant who received the highest dose of POLB 001 had out-of-range ALAT levels, which resolved by day 21.

POLB 001 decreased *ex vivo* TNF secretion, consistent with previously published results (6). Blood samples for *ex vivo* analysis in this study were collected at baseline and on day 6, before administration of the next POLB 001 dose. Compared to the same timepoint (before the next dose was taken) in the previous study, the percentages of inhibition were similar (± 50%–60%).

Administration of ID and IV LPS *in vivo* resulted in consistent, well-detectable inflammatory responses, as expected and in line with previous studies (7, 8, 10, 11). POLB 001 had no meaningful effects on skin erythema or local perfusion after ID LPS administration. However, POLB 001 significantly decreased the attraction of CD3^+^ T cells, classical monocytes, mDCs, T-helper cells, and neutrophils in blister fluid at all dose levels tested. Local LPS-induced increases in IL-1β and TNF were suppressed by the highest dose of POLB 001.

Furthermore, the effect of POLB 001 on systemic inflammatory responses was evaluated using an IV LPS challenge. LPS-driven p38 MAPK phosphorylation in CD14^+^ monocytes was lower in all POLB 001 treatment groups compared with the placebo group, showing a clear dose-dependent effect. Additionally, POLB 001 treatment resulted in lower levels of circulating cytokines (IL-6, IL-8, IL-10, and TNF) and reduced CRP levels following the IV LPS challenge.

The p38 MAPK inhibitors RWJ-67657 and BIRB 796 BS were evaluated in healthy volunteers following an IV LPS dose of 4 ng/kg (1, 12–14). Consistent with our study, both inhibitors suppressed the LPS-induced increase of TNF, IL-6, and IL-8. In addition, both reduced the LPS-induced increase in temperature, and RWJ-67657 also reduced the LPS-induced increase in blood pressure and modulated leukocyte functions. Greater fluctuations in vital signs were observed in these studies compared with our study, which can be attributed to the fourfold higher LPS dose. PF-03715455, PH-797804, and AZD7624 were also shown to inhibit plasma cytokines and neutrophilic airway inflammation following an inhaled LPS challenge in healthy volunteers (15, 16).

Over 20 other p38 MAPK inhibitors have been developed and evaluated in clinical trials across different patient populations. Several have shown (early) evidence of clinical utility, including neflamapimod, pexmetinib, and PH-797804. Neflamapimod demonstrated multiple positive effects in patients with mild Alzheimer’s disease (AD) and in patients with dementia with Lewy bodies and will be further investigated in phase 2b clinical trials for both indications (17, 18). In combination with nivolumab and/or ipilimumab, pexmetinib elicited disease control in a number of PD-(L)1-refractory patients with advanced solid tumours in a phase 1b study (19). PH-797804 demonstrated improvements in lung function and dyspnoea in a phase II study in patients with moderate to severe chronic obstructive pulmonary disease (COPD) (20). Other p38 MAPK inhibitors, such as dilmapimod, ralimetinib, losmapimod, BMS-582949, VX-702, pamapimod, and LY3007113, were evaluated in patients at risk for acute respiratory distress syndrome (ARDS), patients with neuropathic pain, (advanced) cancer, arterial inflammation, or rheumatoid arthritis (RA), but were less successful, or their clinical development was not progressed (21–33).

POLB 001 is in clinical development for the prevention of cancer-immunotherapy-induced CRS. CRS is a common adverse event following CAR T-cell therapies and bispecific antibody therapies. It is characterised by fever, tachypnoea, headache, tachycardia, hypotension, rash, and/or hypoxia caused by the release of cytokines. CRS can progress rapidly, with potentially life-threatening effects, and requires close observation, management, and early intervention. The significant risk of CRS is one of several systemic barriers to wider access to CAR T-cell and T-cell-engaging bispecific antibodies, increasing the cost of treatment and limiting access by requiring a period of inpatient care or close monitoring following treatment. Existing treatment options have been investigated in a prophylactic setting but are less effective at preventing grade ≥ 2 CRS, which requires prompt intervention.

In conclusion, POLB 001 was shown to be safe and effective in decreasing local and systemic LPS-induced inflammation in healthy male volunteers. Moreover, this study provides a solid basis for continuing investigations of POLB 001 in patients with inflammatory conditions.

## Data Availability

Raw data from the current study are available from the corresponding author upon reasonable request.

## Supplementary material

Supplementary Figure 1 Gating strategy of immunophenotyping of blister cells. Expression of cell counts of the different cell subsets present in blister cell fluid are defined in a gating strategy for a whole blood sample (Bsgating). Events were gated in FSC vs. SSC dot plot and debris were excluded. Cells were gated on FSC-Area (A) vs. FSC-Height (H) dot plot to eliminate doublets. To define cell populations, first, live (PI-) CD45^+^ cells are gated. From the leukocyte gate, T cells are identified as CD3^+^ (total), and further subdivided into CD4^+^ (T helper, Th) and CD8^+^ (T cytotoxic, Tc). Next, total HLA^-^DR^+^ cells are gated. From HLA^-^DR^+^ gating, B cells are CD19^+^. From the CD19^-^ downstream gate monocyte subsets are further characterised as: CD14^+^CD16^-^ (classical), CD14^+^CD16^+^ (intermediate) and CD14^-^CD16^+^ (non-classical). From the HLA^-^DR^+^, CD14^-^ CD16^-^ cells, dendritic cells are defined as CD11c^+^ for myeloid DCs (mDCs) and CD123^+^ for plasmacytoid DCs (pDCs). Neutrophils are identified as SSChi, CD16^+^, CD66b^+^. Natural killer (NK) cells are identified from total leukocytes as CD56^+^. FSC, forward scatter; SSC, side scatter.

Supplementary Figure 2 Gating strategy for determining phosphorylation levels of p38 MAPK in *ex-vivo* LPS challenged whole blood. Events were gated in FSC vs. SSC dot plot and debris were excluded. Cells were gated on FSC-Area (A) vs. FSC-Height (H) dot plot to eliminate doublets. To define cell populations, first, live cells (DAPI, VioBlue -) are gated. From the leukocyte gate, monocytes are identified as CD14^+^. Phosphorylation levels of p38 MAPK in all cells - whole blood (live cell gating) and monocytes (CD14^+^) are expressed as percentage (%) and median fluorescence intensity (MFI) of p38 MAPK (PE).

Supplementary Figure 3 *Ex vivo* cytokine release measured in Day 6 samples compared to baseline for all treatment groups: **(A)** IL-1β, **(B)** IL-6, **(C)** IL-8, and **(D)** TNF. TNF, tumour necrosis factor; IL, interleukin.

Supplementary Figure 4 Overview of skin blister fluid analysis by flow cytometry following intradermal LPS administration: **(A)** Total immune cells, **(B)** NK cells, **(C)** non-classical monocytes, **(D)** intermediate monocytes, **(E)** B cells, and **(F)** pDCs. Data are expressed as means with SD. ID, intradermal; LPS, lipopolysaccharide; NK, natural killer; pDCs, plasmacytoid dendritic cells.

Supplementary Figure 5 Circulating leukocyte subset levels during the IV LPS challenge: **(A)** neutrophils and **(B)** monocytes. Data are expressed as means with SD. IV, intravenous; LPS, lipopolysaccharide.

Supplementary Table 1 Antibodies used for immunophenotyping of blister cells and to measure phosphorylation levels of p38 MAPK.

Supplementary Table 2 Treatment-emergent adverse events by preferred term by treatment group (including adverse events reported during the intradermal and intravenous LPS challenge).
