# Supplementary figures and images for: POLB 001, a p38 MAPK inhibitor, decreases local and systemic inflammatory responses following in vivo LPS administration in healthy volunteers: a randomised, double-blind, placebo-controlled study
Source: Front Immunol. 2026 Jan 23;16:1684307. doi: 10.3389/fimmu.2025.1684307 (PMC12877788; doi:10.3389/fimmu.2025.1684307)

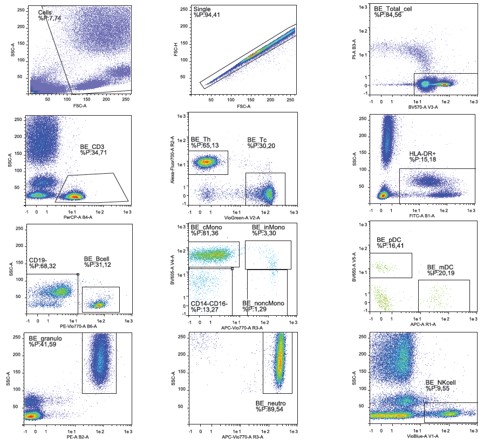

Supplement: Supplementary Figure 1 — Gating strategy of immunophenotyping of blister cells. Expression of cell counts of the different cell subsets present in blister cell fluid are defined in a gating strategy for a whole blood sample (Bsgating). Events were gated in FSC vs. SSC dot plot and debris were excluded. Cells were gated on FSC-Area (A) vs. FSC-Height (H) dot plot to eliminate doublets. To define cell populations, first, live (PI-) CD45+ cells are gated. From the leukocyte gate, T cells are identified as CD3+ (total), and further subdivided into CD4+ (T helper, Th) and CD8+ (T cytotoxic, Tc). Next, total HLA-DR+ cells are gated. From HLA-DR+ gating, B cells are CD19+. From the CD19- downstream gate monocyte subsets are further characterised as: CD14+CD16- (classical), CD14+CD16+ (intermediate) and CD14-CD16+ (non-classical). From the HLA-DR+, CD14- CD16- cells, dendritic cells are defined as CD11c+ for myeloid DCs (mDCs) and CD123+ for plasmacytoid DCs (pDCs). Neutrophils are identified as SSChi, CD16+, CD66b+. Natural killer (NK) cells are identified from total leukocytes as CD56+. FSC, forward scatter; SSC, side scatter. [file Image1.jpeg]

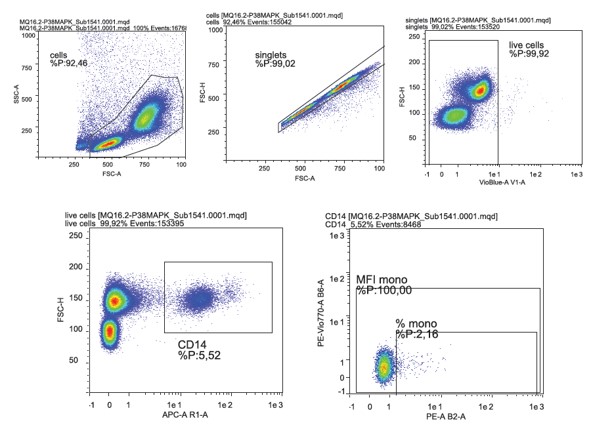

Supplement: Supplementary Figure 2 — Gating strategy for determining phosphorylation levels of p38 MAPK in ex-vivo LPS challenged whole blood. Events were gated in FSC vs. SSC dot plot and debris were excluded. Cells were gated on FSC-Area (A) vs. FSC-Height (H) dot plot to eliminate doublets. To define cell populations, first, live cells (DAPI, VioBlue -) are gated. From the leukocyte gate, monocytes are identified as CD14+. Phosphorylation levels of p38 MAPK in all cells - whole blood (live cell gating) and monocytes (CD14+) are expressed as percentage (%) and median fluorescence intensity (MFI) of p38 MAPK (PE). [file Image2.jpeg]

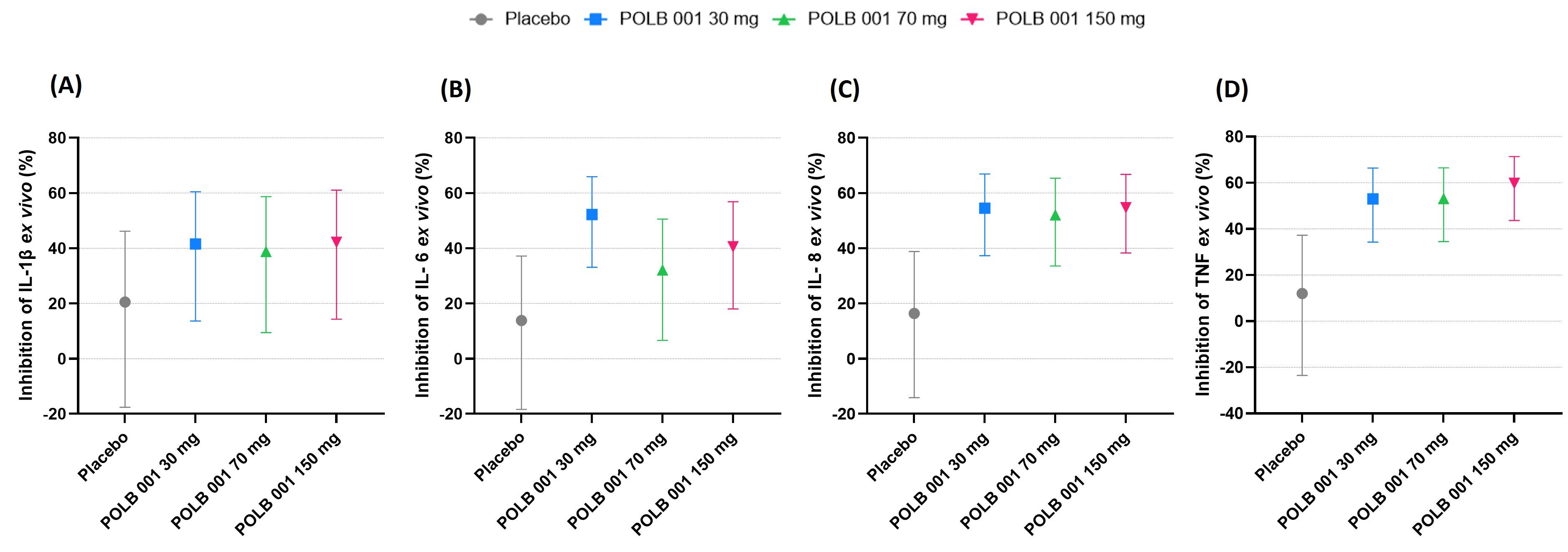

Supplement: Supplementary Figure 3 — Ex vivo cytokine release measured in Day 6 samples compared to baseline for all treatment groups: (A) IL-1β, (B) IL-6, (C) IL-8, and (D) TNF. TNF, tumour necrosis factor; IL, interleukin. [file Image3.jpg]

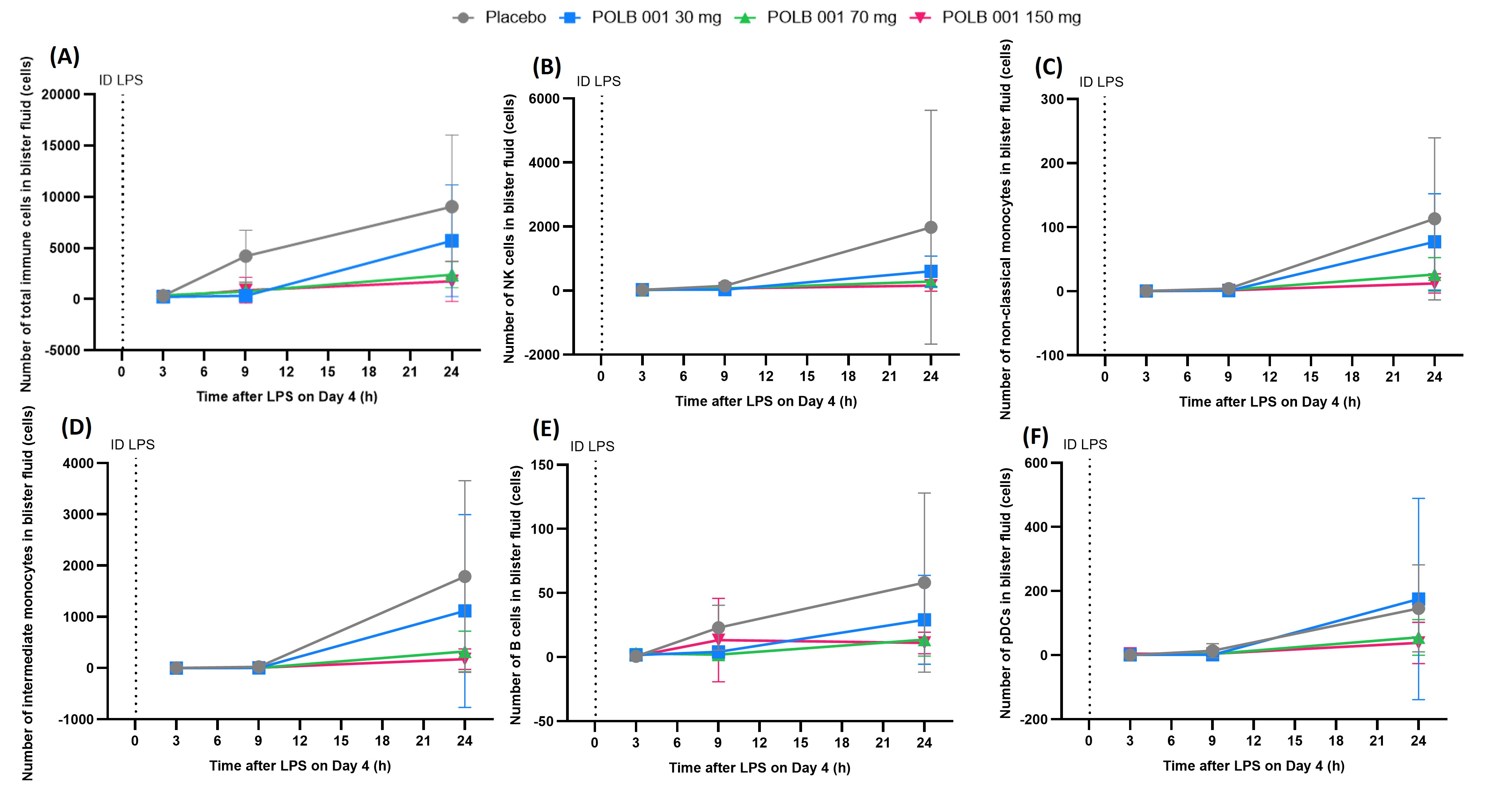

Supplement: Supplementary Figure 4 — Overview of skin blister fluid analysis by flow cytometry following intradermal LPS administration: (A) Total immune cells, (B) NK cells, (C) non-classical monocytes, (D) intermediate monocytes, (E) B cells, and (F) pDCs. Data are expressed as means with SD. ID, intradermal; LPS, lipopolysaccharide; NK, natural killer; pDCs, plasmacytoid dendritic cells. [file Image4.jpg]

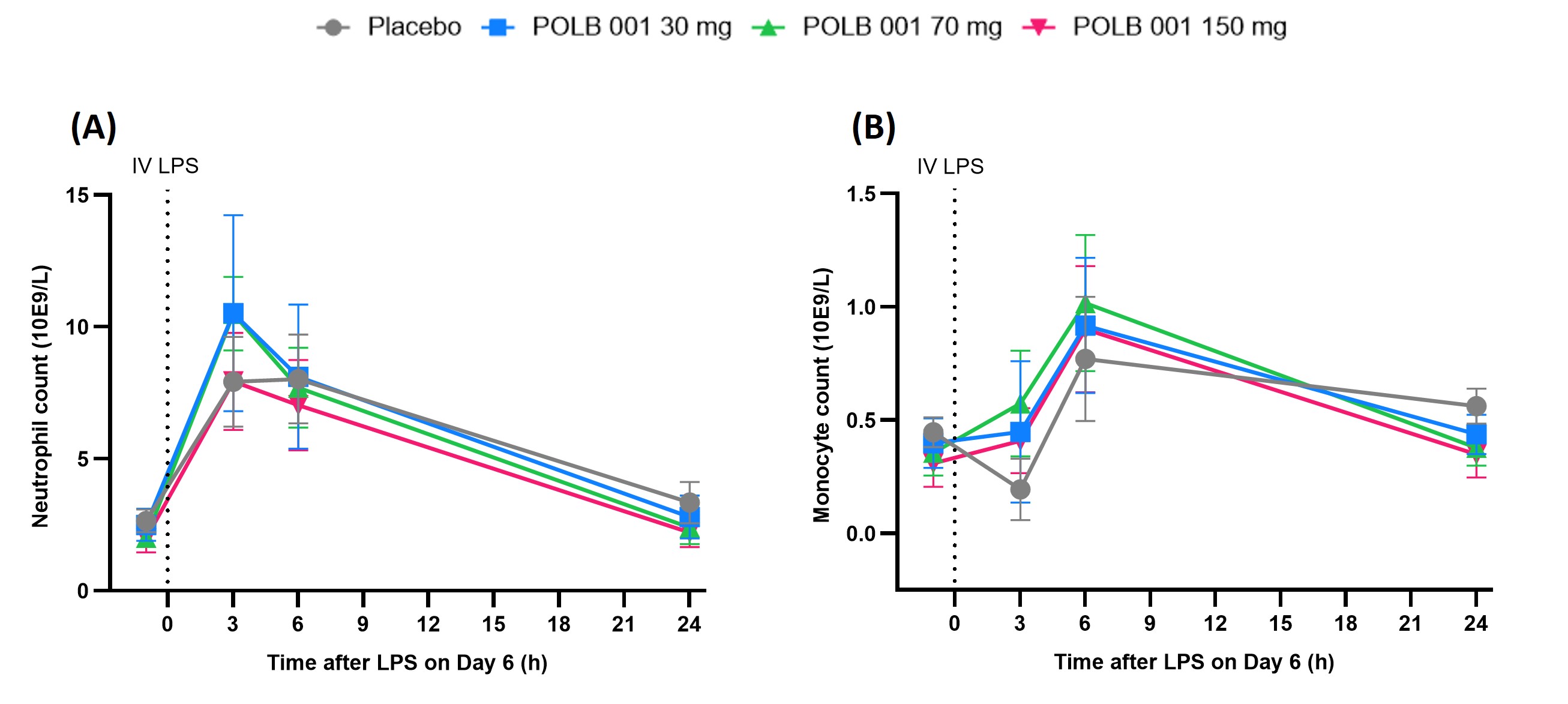

Supplement: Supplementary Figure 5 — Circulating leukocyte subset levels during the IV LPS challenge: (A) neutrophils and (B) monocytes. Data are expressed as means with SD. IV, intravenous; LPS, lipopolysaccharide. [file Image5.jpg]

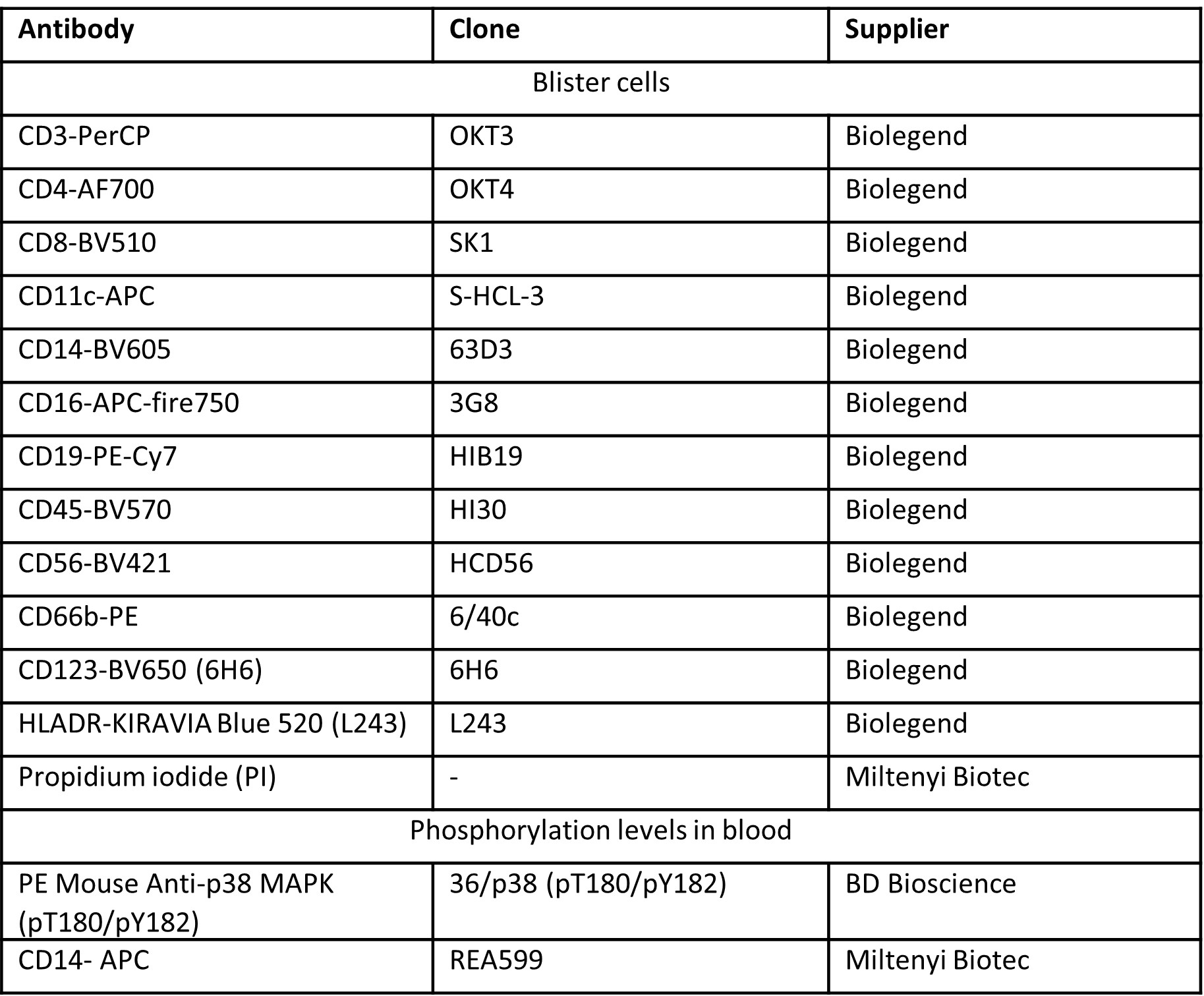

Supplement: Supplementary Table 1 — Antibodies used for immunophenotyping of blister cells and to measure phosphorylation levels of p38 MAPK. [file Image6.jpeg]

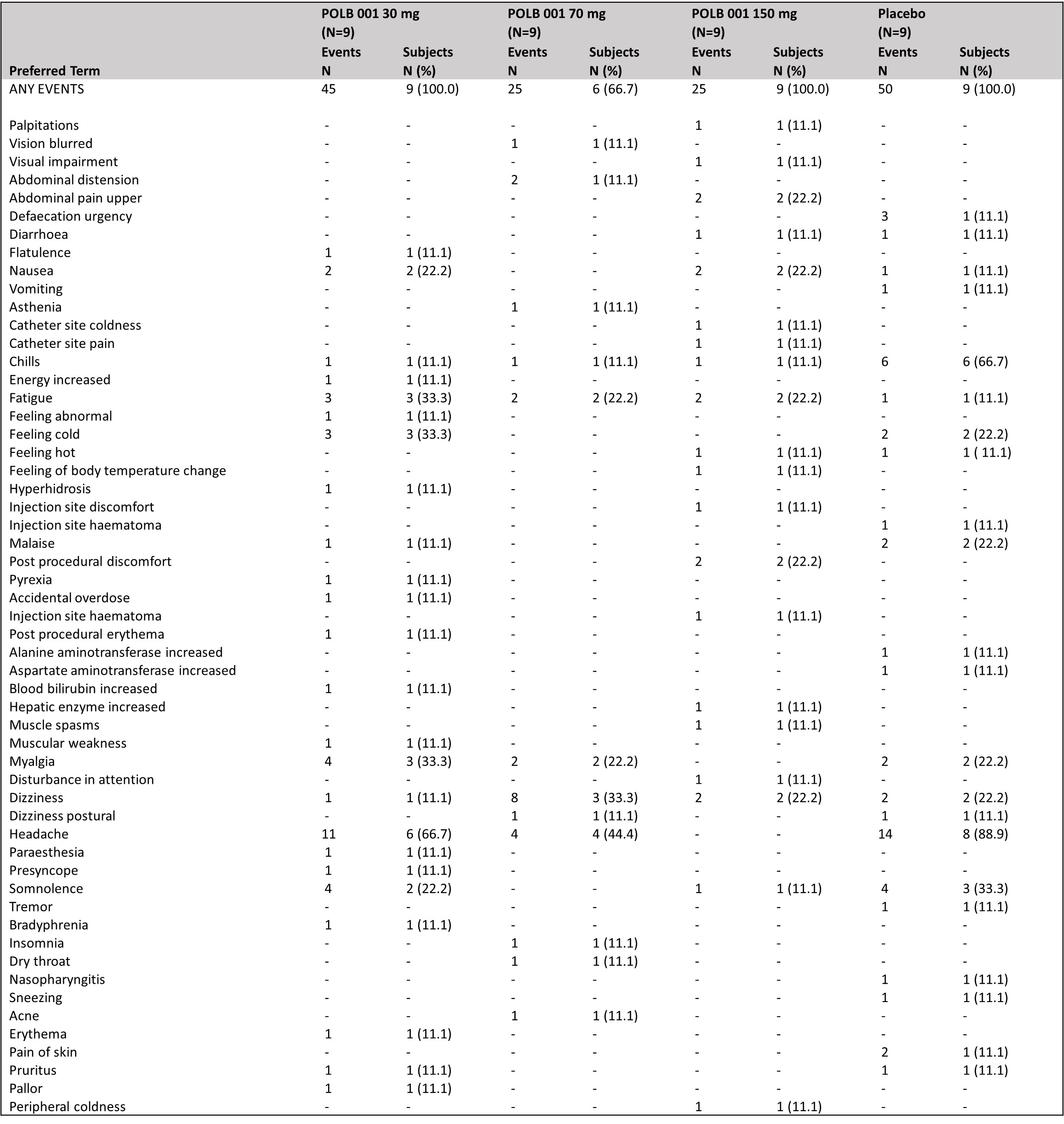

Supplement: Supplementary Table 2 — Treatment-emergent adverse events by preferred term by treatment group (including adverse events reported during the intradermal and intravenous LPS challenge). [file Image7.jpeg]
